# Transcriptome analysis identifies genes involved in adventitious branches formation of *Gracilaria lichenoides in vitro*

**DOI:** 10.1038/srep17099

**Published:** 2015-12-11

**Authors:** Wenlei Wang, Huanqin Li, Xiangzhi Lin, Shanjun Yang, Zhaokai Wang, Baishan Fang

**Affiliations:** 1 College of Biochemistry and Engineering, Xiamen University, Xiamen 361005, China; 2 Engineering Research Center of Marine Biological Resource Comprehensive Utilization, Third Institute of Oceanography, State Oceanic Administration, Xiamen 361005, China; 3 College of Ocean and Earth Sciences, Xiamen University, Xiamen 361005, China

## Abstract

Tissue culture could solve the problems associated with *Gracilaria* cultivation, including the consistent supply of high-quality seed stock, strain improvement, and efficient mass culture of high-yielding commercial strains. However, STC lags behind that of higher plants because of the paucity of genomic information. Transcriptome analysis and the identification of potential unigenes involved in the formation and regeneration of callus or direct induction of ABs are essential. Herein, the CK, EWAB and NPA *G. lichenoides* transcriptomes were analyzed using the Illumina sequencing platform in first time. A total of 17,922,453,300 nucleotide clean bases were generated and assembled into 21,294 unigenes, providing a total gene space of 400,912,038 nucleotides with an average length of 1,883 and N 50 of 5,055 nucleotides and a G + C content of 52.02%. BLAST analysis resulted in the assignment of 13,724 (97.5%), 3,740 (26.6%), 9,934 (70.6%), 10,611 (75.4%), 9,490 (67.4%), and 7,773 (55.2%) unigenes were annotated to the NR, NT, Swiss-Prot, KEGG, COG, and GO databases, respectively, and the total of annotated unigenes was 14,070. A total of 17,099 transcripts were predicted to possess open reading frames, including 3,238 predicted and 13,861 blasted based on protein databases. In addition, 3,287 SSRs were detected in *G.lichenoides*, providing further support for genetic variation and marker-assisted selection in the future. Our results suggest that auxin polar transport, auxin signal transduction, crosstalk with other endogenous plant hormones and antioxidant systems, play important roles for ABs formation in *G. lichenoides* explants *in vitro*. The present findings will facilitate further studies on gene discovery and on the molecular mechanisms underlying the tissue culture of seaweed.

Global demand for seaweed resources has increased significantly because of their emergent use as sources of phycocolloids, biopharmaceuticals, nutraceuticals and biofuels. Intertidal seaweed species could survive despite they often suffer from high temperature, strong light, sever desiccation stress^1^,^2^,^3^,^4^,^5^. For example, *G. lichenoides* has a high growth rate and can tolerate high temperatures^4^. *Gracilaria* is ecologically and economically important red seaweed that is used mainly for the production of phycocolloids. Approximately 60–80% of the world’s agar production depends on *Gracilaria*^6^,^7^,^8^. Some species of *Gracilaria* have been identified as a robust and effective alternative for sea nutrient bioremediation^9^ or soil amelioration^10^. *G. lichenoides* could efficiently absorb inorganic nitrogen and inorganic phosphate. Meanwhile, it also can be used as food for abalone or other aquacultured animals^9^.

Seaweed tissue culture (STC) is an important tool for micropropagation. It facilitates the production of a large number of seedlings for seaweed cultivation and genetic engineering^11^ within a short period of time, eliminating the need for laborious seedling collection from natural beds^12^. STC is unique among *in vitro* culture techniques; however, progress in the field is lagging far behind that of land plants because of a lack of genomic information. Callus formation has been reported in some species of Gracilariales, including *G. verrucosa*^13^,^14^, *G. textorii*^15^, *G. vermiculophylla*^16^, *G. tenuifrons*^17^, *G. chilensis*^18^, *G. tenuistipitata*, *G. perplexa*^19^, *G. corticata*^20^, and *G. domingensis*^21^. However, in most species, callus formation does not occur often and cannot be controlled easily, as in higher plants through the introduction of exogenous plant hormones. Despite the addition of auxins and cytokinins (CTK) alone or in combination, regeneration from callus is difficult. Furthermore, the main morphological response of explants of Gracilariales to *in vitro* culture is the direct production of adventitious branches (ABs) within a shorter period of time than that required for callus formation as a result of wounding, as reported for *G. blodgettii*, *Gracilariopsis lemaneiformis*, *G. tenuistipitata*, *G. asiatica*, *G. vermiculophylla*, *G. domingensis*, and *G. changii*^21^,^22^,^23^,^24^,^25^. Similar results were reported for *Gelidiales*, *Halymeniales*, *Ceramiales* and *Gigartinales*^26^,^27^,^28^, which illustrates cell totipotency and is an alternative morphological response that could be used for seedling production instead of thallus regeneration from callus. However, few studies have investigated the mechanisms of ABs formation, and improving our understanding of the process of ABs formation and development in seaweed species would be of value.

Studies addressing the role of exogenous plant hormones in the formation of ABs in seaweed explants have reported inconsistent results. The differential sensitivity of explants to plant hormones might be due to an inherent regulatory mechanism rather than the concentration of plant growth regulators^29^. Aguirre-Lipperheide *et al.* showed that the direct regeneration of micropropagules from the explants of red seaweed is regulated by internal factors rather than exogenous plant hormones or other external factors^30^. Endogenous plant hormones, which play a significant role in almost every aspect of plant growth and development, have been detected in green, brown and red seaweeds^31^. Nevertheless, the evidence for a more direct physiological and developmental role of endogenous plant hormones in algae is limited and inconclusive. Furthermore, auxin is the only endogenous hormone involved in polar transport. The establishment of an auxin gradient and polar auxin transport (PAT) mediated by PIN1, a subfamily of ABC transporters (ABCBs), is essential for callus induction and somatic embryogenesis in model land plants such as *Arabidopsis*^32^,^33^,^34^. Understanding the role of endogenous hormones in the regulation of seaweed growth and development is important to improve tissue culture techniques.

Sequencing technology has developed rapidly in recent years, and transcriptome analysis has become increasingly powerful. RNA sequencing (RNA-seq) enables the rapid and comprehensive analysis of plants and other organisms^35^,^36^. The intensive study of transcriptome sequencing has provided a cost-effective method for the qualitative and quantitative analysis of gene transcripts in many non-model plant species such as *Gracilaria*^37^. In the present study, we used the Illumina sequencing platform to examine the transcriptional profiles of samples of *G. lichenoides* exposed to different treatments, including a control(CK), explants with adventitious branches (EWAB), and explants treated with N-1-naphthylphthalamic acid (NPA), a PAT inhibitor.

## Materials and Methods

### G. lichenoides culture

*G. lichenoides* thalli were collected from the Dongshan coast, Fujian Province. The fresh samples were cleared of mud and epiphytes using a soft brush and filtered seawater, and cultured in tanks with f/2 medium^38^ at 25 ± 1 °C under cool-white fluorescent illumination (30 μmol photons m^−2^ s^−1^) in a12:12 light:dark (L:D) cycle. The water inside the tanks was agitated with constant aeration and the culture medium was replaced every 3 days.

### Tissue culture and sample collection

Healthy thalli of *G. lichenoides* were examined under a microscope, and those with the least contamination of epiphytes were selected and sterilized according to the methodology described by Shen *et al*^39^. Briefly, thalli were washed three times in autoclaved seawater with a writing brush, three times in an ultrasonic cleaner (Branson, Beijing, China) and three times in 0.7% potassium iodide (KI).

The sterilized thalli were cut into short explants (5–7 mm) using a sterile surgical blade and inoculated in a 200-mL culture vessel for liquid media with f/2 medium (21 ± 1 °C, 30 μmol photons m^−2^ s^−1^ irradiance, 14-h: 10-h, light-dark cycle), which was replaced every 3 days. The effect of auxins (indole-3-acetic acid, IAA) and NPA (1 and 100 μM, respectively) and their combination on callus formation or AB induction in *G. lichenoides* explants was determined. A treatment control (without IAA or NPA) was prepared simultaneously. After exposure to the different conditions, the samples were subjected to extraction of RNA.

### RNA extraction,de novo transcriptome assembly and annotation

The transcriptional profiles of the differently treated samples of *G. lichenoides* (CK, EWAB and NPA) were examined using the Illumina sequencing platform. Total RNA was extracted according to Method 3 as described by Chan *et al*^40^. The extracted RNA was qualified and quantified using a Nanodrop ND-1000 Spectrophotometer (Nanodrop Technologies,Wilmington, DE, USA) and all samples showed a 260/280 nm ratio between 1.9 and 2.1 with no sign of degradation. The construction and sequencing of cDNA libraries were performed by BGI (Shenzhen, China) on an Illumina HiSeq 2000 platform (San Diego, CA, USA) in accordance with the manufacturer’s instructions.

A filter was used to remove low-quality reads, which were defined as those with adaptor or ambiguous bases (‘N’ < 5%), as well as all reads with nucleotides that had Phred^41^ quality scores <20, where a Phred score of 20 corresponds to a 1% expected error rate. Ultimately, clean reads were obtained by this filtering process and assembled into unigenes with the Trinity program^42^ (release-20130225, http://trinityrnaseq.sourceforge.net/). Since there is no reference genome of *G. lichenoides*, a k-mer value cutoff of 25 was used after removing redundant nucleotide sequences by Tgicl (v2.1, http://sourceforge.net/projects/tgicl/files/tgicl%20v2.1/).

All the unigenes were used for functional annotation by BLASTX against various protein databases, such as non-redundant protein (Nr) databases, the Swiss-Prot database, the Kyoto Encyclopedia of Genes and Genomes (KEGG) database, and the Cluster of Orthologous Groups (COG) database, as well as the NCBI non-redundant nucleotide sequence (Nt) database with an E value cutoff of 10^−5^ 43, and the best aligning results were selected to annotate the unigenes. If the aligning results from different databases were in conflict with each other, the results from the Nr database were preferentially selected, followed by the Swissprot, KEGG and COG databases. CDS (coding sequence) was obtained by blasting against these four databases, as well as by using ESTScan^44^. Gene ontology (GO) annotation for the unigenes was based on the best alignment of Swissprot obtained using the Blast2GO program^45^, and WEGO software^46^ was then used to plot the GO annotation results. Pathway assignments were performed according to the KEGG pathway database^47^.

### Differential expression analysis of unigenes

The specifically expressed genes were screened from the distinct *G. lichenoides* explants by differential expression analysis. The expression levels of unigenes were calculated using reads per kilobase transcriptome per million mapped reads (RPKMs) to eliminate the effect of gene length and sequencing level on the calculation of gene expression^48^. The calculated results were directly used to analyze differences in gene expression levels between the different samples using the statistical comparison method^49^. The results of multiple hypothesis testing were corrected using the false discovery rate (FDR) control method, and the ratio of RPKMs was converted to fold-changes in the expression of each gene in two samples simultaneously. Significant differentially expressed genes were screened according to the FDR threshold of ≤0.001 and the absolute value of |log_2_Ratio| ≥1. When differentially expressed genes were identified, all significantly differentially expressed genes (DEGs) were mapped to the KEGG database to conduct pathway enrichment analysis to identify significantly enriched metabolic pathways or signal transduction pathways with differentially expressed genes using hypergeometric tests. The calculated q-values were subjected to Bonferroni correction, with a corrected q-value ≤0.05 as a threshold. Unigenes were annotated with the NR, NT, Swiss-Prot, KEGG, COG and GO databases, and the number of unigenes annotated with each database was counted.

### Identification of simple sequence repeats

All unique sequences were searched using microsatellite search module (MISA, http://pgrc.ipk-gatersleben.de/misa/misa.html) of Primer 3 (http://www.onlinedown.net/soft/51549.htm) for simple sequence repeats (SSRs) with 2–6 bp repeats as described by Thiel *et al*^50^. Di-, tri-, tetra-, penta- and hexa-nucleotide sequences repeated more than six, five, five, four and four times, respectively, were captured as SSRs.

## Results

### Tissue culture of *G. lichenoides*

**Figure 1:**
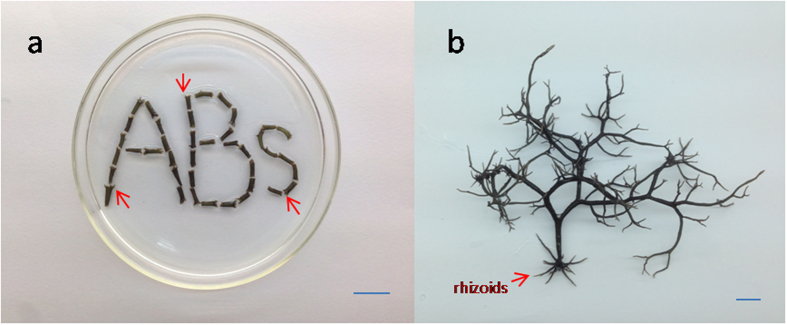
Regeneration of segments of *G. lichenoides in vitro*. (**a**). ABs formation from explants of *G. lichenoides in vitro* (red arrow indicating ABs). Bar: 1 mm. (**b**). Thallus showing rhizoid growth from ABs after 4 months. Bar: 1 mm.

In all experiments, the main morphogenetic response of the explants of *G. lichenoides in vitro* was the production of direct ABs from the cortex within 8 days rather than callus formation as a result of wounding. The rate of ABs formation was 94.9%. ABs became mature with rhizoids 4 months after leaving cut segments (Fig. 1). NPA inhibited ABs formation in the explants of *G. lichenoides*, and this effect was not reversed by the addition of IAA. However, ABs formed after transfer of the explants to normal medium.

### Illumina sequencing, assembly and sequence analysis

A total of 17,922,453,300 nucleotide (nt) paired-end reads with a read length of 100 bp were obtained conveniently and efficiently using the three cDNA libraries, which were sequenced with Illumina HiSeq™ 2000. The G + C percentages were all approximately 52% (Table 1). The percentage of Q20 bases (those with a base quality >20 and an error rate <0.01) was higher than 99%, indicating the high quality of the raw sequencing reads of the sample (Table 1). After stringent quality assessment and data filtering performed using the Trinity *de novo* assembly program, the next-generation short-read sequences were assembled into 21,294 unigenes, which represented putative protein-coding transcripts. These consisted of the partial non-overlapping coding regions of *G. lichenoides*, with an N50 [length of the smallest transcripts in the set containing the fewest (largest)] length of 5,055 nt and a mean length of 1,883 nt. An overview of the assembly results is provided in Table 2.

### Functional annotation of the transcriptome

Regarding the BLAST results, 13,724 (97.5%), 3,740 (26.6%), 9,934 (70.6%), 10,611 (75.4%), 9,490 (67.4%), and 7,773 (55.2%) unigenes were annotated to the NR, NT, Swiss-Prot, KEGG, COG, and GO databases, respectively, and the total of annotated unigenes was 14,070. In addition, 3,287 SSRs were detected, providing further support for genetic variation and marker-assisted selection in the future.

**Figure 2:**
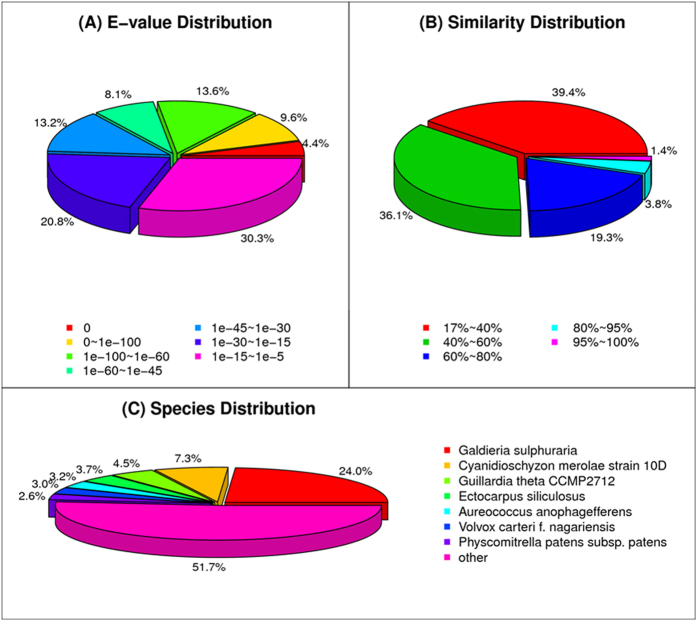
Results of the similarity search of the assembled sequences against the Nr database. (**a**) E value distribution of blast hits for each unique sequence with E value ≤10^−5^. (**b**) Similarity distribution of the top blast hits for each sequence. (**c**) Species distribution of the total homologous sequences with E value ≤10^−5^ The first hit of each sequence was used for statistical analysis.

In the Nr annotation, 6,709 sequences had perfect matches with E values <10^−45^ and 5.2% of the matches had a similarity over 95% (Fig. 2a,b). The species distribution showed that 30.0% had top matches to the *Galdieria sulphuraria* genes (Fig. 2c). The next two top matching species were the *Cyanidioschyzon merolae* strain 10D and *Guillardia theta* CCMP2712, accounting for 4.5 and 3.7%, respectively. Only 1.16% of the sequences showed matches to *Gracilaria*.

**Figure 3:**
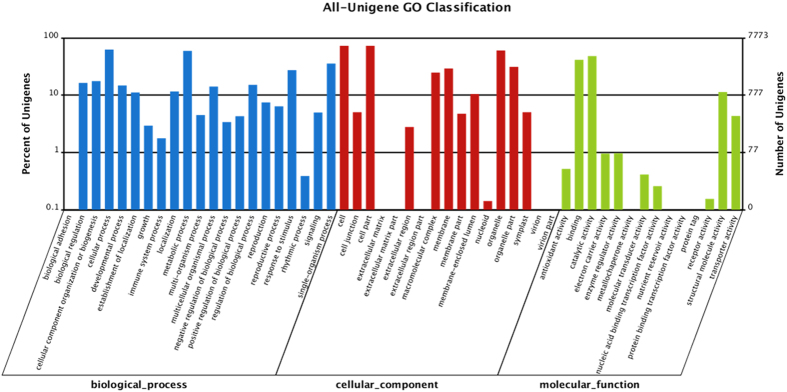
Gene ontology classification of all annotated transcripts in the *G. lichenoides* transcriptome.

GO analysis was conducted on the annotated 58,214 unique sequences using the Blast2GO program. The unique sequences were successfully assigned to biological processes, cellular components, and a molecular function GO category, respectively (Fig. 3). The most abundant were “cellular processes” (4,856 members) and metabolic processes (4,606 members) in the biological process category, and cells (5,651 members), cell parts (5,650 members) and organelles (4,677 members) for the cellular component category. Most of the sequences were classified into catalytic activity (3,744 members) and binding (3,211 members) functions in the molecular function category. Additional information derived from the GO annotation was as follows: of the unigenes analyzed, 2,123 were annotated into the response to stimulus category, 580 into reproduction, 496 into reproductive processes, 336 into transporter activity, and 40 into antioxidant activity, which may provide an explanation for the *in vitro* regeneration mechanism of macroalgae explants.

**Figure 4:**
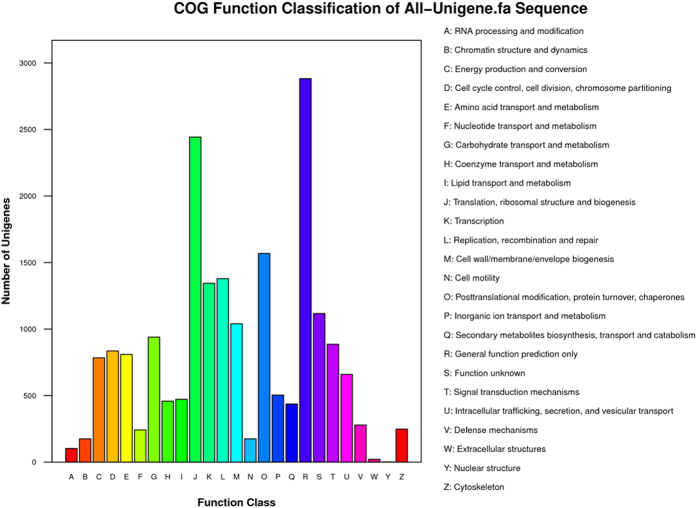
Clusters of Orthologous Groups (COG) functional classification of *G. lichenoides* transcriptome sequences.

To further evaluate the completeness of our transcriptome library and the effectiveness of our annotation process, all annotated unigenes were aligned to the COG database to predict and classify possible functions. In total, 9,490 sequences had a COG classification. Among the 25 COG categories, “general function prediction only” represented the largest group (2,882 members), followed by “translation, ribosomal structure and biogenesis” (2,443 members) and “post-translational modification, protein turnover, chaperones” (1,568 members) (Fig. 4).

The KEGG pathway database records the networks of molecular interactions in the cell and variants specific to particular organisms. Pathway-based analyses are important to further understand the biological functions and interactions of genes. In total, 10,611 sequences were assigned to 126 KEGG pathways. The pathways with the most representation among the unique sequences were the metabolic pathways (2,654 unigenes), followed by Ribosome (1,233 unigenes) and biosynthesis of secondary metabolites (1,089 unigenes).A hallmark of dedifferentiation and the subsequent formation of ABs is the ability to control cell division and cell wall accumulation associated with polysaccharide metabolism. This is associated with the expression of the corresponding hydrolytic enzymes and their accumulation of storage reserves and secondary metabolites. Furthermore, some important pathways related to plant growth, development, morphogenesis and response to stress stimuli, including ABC transporters (222 unigenes), ubiquitin-mediated proteolysis (152 unigenes), glutathione metabolism (119 unigenes), peroxisome(106 unigenes), carbon fixation in photosynthetic organisms (92 unigenes), phosphatidylinositol signaling system (67 unigenes), plant hormone signal transduction(61 unigenes), zeatin biosynthesis(38 unigenes) and others, have also been annotated successfully.

### Global analysis of differentially expressed genes

**Figure 5:**
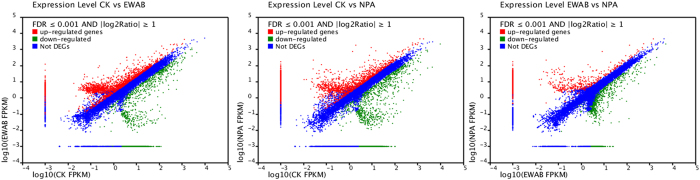
Gene expression changes in different treated groups (The genes were classified into three classes. Red genes are up-regulated that gene expression of right sample is larger than left sample. Green genes are down-regulated that gene expression of left sample is larger than right sample. Blue genes are not differentially expressed genes. The horizontal coordinates is the expression level of right and the vertical coordinates is the expression level of left sample).

RSEM software was applied to map the clean reads in different libraries to the corresponding assembled consensus sequences. A strict binomial distribution algorithm was used to identify DEGs, considering the differences in library size for the differential selection of DEGs among libraries. Pairwise comparisons between the above three groups (CK, EWAB and NPA) were performed for differential expression analysis (Fig. 5). To gain an understanding of the genes expressed in this time course study, we selected genes that were differentially expressed between two groups by log_2_ (fold change) >10.

In the CK/EWAB pairwise comparison, 9,525 genes showed differential expression, including 2,800 up-regulated genes and 6,725 down-regulated genes in EWAB. In the CK/NPA pairwise comparison, there were 9,891 DEGs, including 3,651 up-regulated genes and 6,240 down-regulated genes in NPA (Fig. 5). GO term enrichment analysis was performed to evaluate significantly over-represented GO terms in the differentially expressed genes, which resulted in a total of 49 GO terms with a p-value <0.05 and FDR <0.01. These GO terms included functions and processes such as reproduction, reproductive processes (Fig. 3), auxin polar transport, and response to auxin stimulation (Table 3). Furthermore, 4,087 (42.9%) and 4, 217 (42.6%) unigenes were annotated to the KEGG pathway in CK/EWAB and CK/NPA, respectively. The majority of those annotated genes between CK/EWAB and CK/NPA had similar expression patterns and were related to plant growth and development, cell vegetation and differentiation, and response to stimulus, mainly including ABC transporters, ubiquitin-mediated proteolysis, plant hormone signal transduction and other pathways (Table 4). The process of *in vitro* regeneration of explants from macroalgae was complex.

Between EWAB and NPA, 4,264 DEGs were discovered, of which 2,688 genes were up-regulated and 1,576 were down-regulated in NPA (Fig. 5). GO term enrichment analysis included functions and processes such as auxin polar transport (one up-regulated unigene), auxin-mediated signaling pathways (two up- and two down-regulated unigenes), response to the auxin stimulus(six up- and two down-regulated unigenes), and auxin biosynthetic processes(one up-regulated unigene) (Table 3). In addition, 2,108 (49.4%) unigenes were annotated to the KEGG pathway, mainly including the ABCB1 family of ABC transporters (five up- and one down-regulated unigenes), ubiquitin-mediated proteolysis (10 up- and one down-regulated unigenes), plant hormone signal transduction (10 up-regulated unigenes) and antioxidant systems (18 up- andthree down-regulated unigenes) (Table 4). Given the known role of auxin in regulating plant embryogenesis, the competence for AB induction might be the result of regulated auxin responses of these cells. These annotations provide a valuable resource for investigating specific processes, functions, and pathways and allow for the identification of novel genes involved in these pathways.

### Heterogeneous SSR detection

**Figure 6:**
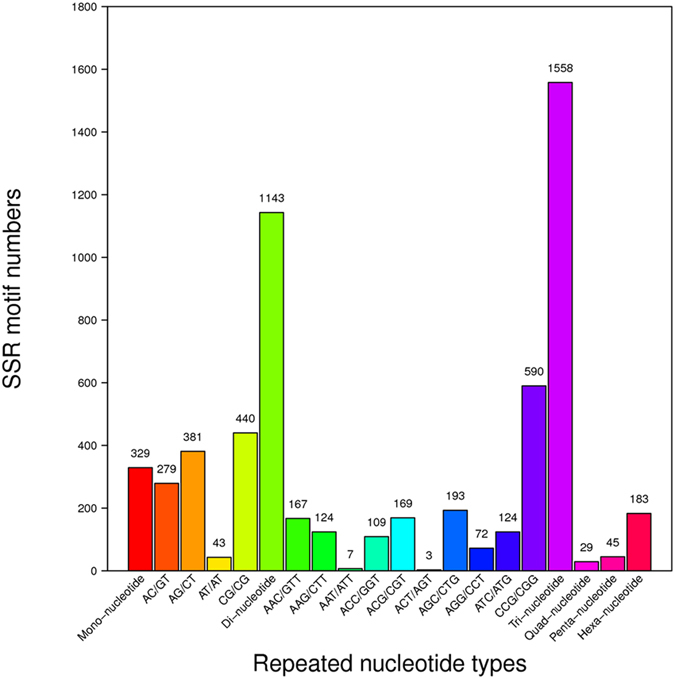
Summary of cSSR search results in *G. lichenoides*.

SSR detection performed using MISA software with unigenes as a reference resulted in the identification of 3,287 high-quality SSRs. This dataset included 1,143 SSRs with two nucleotides repeated more than six times, 1,558 SSRs with three nucleotides repeated more than five times, and 228 SSRs with five or more nucleotides repeated more than four times. The SSR discovery revealed that tri-nucleotide repeats are the most abundant because any addition or reduction in repeat numbers would not cause frame shift mutations. Among TriNR, GCG was the most common motif, followed by GGC and CGC. The most abundant motifs were GC in DiNR, GCGT in TetraNR, CCAAA in PentaNR, and CCGCGT in HexaNR (Fig. 6).

## Discussion

In the present study, although callus induction was not observed, ABs were successfully formed on the cut surfaces of *G. lichenoides* explants in response to most treatments, showing the capacity for regeneration after wounding. Similar results were obtained in *Gelidiales*, *Halymeniales*, *Ceramiales* and *Gigartinales*, and other species of *Gracilaria*^25^,^26^,^27^,^28^. Huang and Fujita reported that callus was not induced from explants of *G. textorii* using IAA^15^. Collantes *et al.* revealed that the addition of IAA and kinetin to PES medium did not promote callus formation in *G. chilensis* explants as a result of wounding^18^. Ramlov *et al.* showed that IAA (50.0 μM), 2, 4-dichlorophenoxyacetic acid (2, 4-D, 0.5 μM) and 6-Benzylaminopurine (6-BA) all stimulated direct plant regeneration, which was suitable for the micropropagation of *G. domingensis*^21^. Yeong *et al.* reported that combinations of auxin and CTK enhanced adventitious shoot induction from the cortical zone of the cut surface of explants of *G. changii* directly, without an intervening callus phase^19^. However, Yokoya *et al.* reported that different types of callus were stimulated successfully using different concentrations of IAA, 2, 4-D and kinetin in *G. tenuistipitata* and *G. perplexa*, and that different *Gracilaria* species or even different types of cells responded differently to the plant growth regulators^19^. These studies suggest that the differential sensitivity of macroalgae explants to plant hormones could be attributed to an inherent regulatory mechanism rather than the concentration or combination of plant hormones^29^. In our study, NPA, an inhibitor of PAT, effectively prevented the formation of ABs in *G. lichenoides*, suggesting that PAT plays an important role in the process of regeneration of macroalgae. This was consistent with results obtained in higher plants^32^,^33^,^34^. Moreover, the detection of increases in DNA, RNA and protein content prior to or concomitant with morphological responses indicates changes in gene expression^51^. The development of STC techniques in the near future and their combination with molecular genetics could provide support for the same biotechnological applications used in higher plants in the genomic age^26^,^52^,^53^.

Transcriptome sequencing is one of the most important tools for expression pattern identification and gene discovery^54^,^55^. Here, we used transcriptome sequencing with the Illumina platform to regenerate macroalgae explants *in vitro*. Functional enrichment showed the presence of many regeneration-related genes in the transcriptome (Tables 3 and 4), indicating a complex regeneration system in *G. lichenoides*. The following gene families are potentially valuable for future research:

### Signal transduction pathways associated with auxin and other plant hormones

Auxin plays an important role in dedifferentiation and redifferentiation through the modulated response, transduction, and amplification of the auxin signal leading to the regulation of gene expression. Auxin was previously detected in *Gracilaria sp*^31^. In the present study, 49 genes related to auxin biosynthesis (four transcripts), transport (11 transcripts), metabolism (nine transcripts), response to the auxin stimulus (27 transcripts), auxin response factor (ARF) (one transcript), and ABCB1 (26 transcripts), were differentially expressed during ABs formation (Table 3 and 4).

In general, plant development and physiology depend on a unique, plant-specific process, namely cell-to-cell or polar auxin transport. PAT is controlled by efflux proteins, including PIN and ATP binding cassette type B/P-glycoprotein/multidrug resistance (ABCB/PGP/MDR) proteins. ABCB- and PIN-mediated auxin efflux can function independently and play identical cellular but separate developmental roles^56^. *E.siliculosus*, for instance, has several ABCB efflux IAA transporters that participate in the polarized transport of IAA and stabilize PIN1^57^. ABCB1 and ABCB19 were recently identified as binding proteins of the synthetic auxin-efflux inhibitor, 1-N-Naphtylphthalamic acid (NPA)^58^,^59^,^60^,^61^,^62^. TWISTED DWARF1 (TWD1) binds to NPA, which disrupts the TWD1-ABCB1 interaction^58^,^63^. This disrupts ABCB1 activity, suggesting that TWD1 and ABCB1 represent essential components of the NPA-sensitive-efflux complex^63^. Several lines of evidence suggest that PIN proteins do not themselves act as direct targets of NPA^34^,^62^,^64^,^65^. Here, 26 auxin transport-associated ABCB1 transcripts were found to be differentially expressed. Nineteen ABCB1 transcripts were down-regulated and one transcript was up-regulated in the CK/EWAB group, whereas 22 (five up- and 17 down-regulated) and six (five up- and one down-regulated) ABCB1 transcripts were differentially expressed in the CK/NPA and EWAB/NPA groups, respectively (Table 4). The annotated ABCB1 genes mainly down-regulated, which are direct binding proteins of NPA, during the formation of ABs indicating that they might negatively regulate PAT. By contrast, five ABCB1 unigenes were up-regulated by NPA, indicating that those five genes might be induced by NPA and prevent ABs formation by inhibiting PAT. Unigene11327_All was obviously up-regulated in the CK/EWAB group and down-regulated in the EWAB/NPA group, indicating that this gene might promote PAT. Unexpectedly, influx (AUX1) and efflux (PIN) proteins were not annotated in our study. This could be attributed to NPA may not be strictly specific for PIN proteins. It is thus possible that the auxin efflux transporter of *G. lichenoides*is different from that of higher plants.

ARF, which is a type of transcription factor with B3 DNA binding domains, mediates auxin responses and regulates the expression of auxin-induced genes by binding to auxin response elements (AuxRE) in specific gene promoter regions^66^. Because Aux/IAA proteins can function as active repressors, dimerization between an ARF transcriptional activator bound to an AuxRE and an Aux/IAA protein would result in the repression of auxin response genes in higher plants. When auxin concentrations increase, Aux/IAA repressors are degraded rapidly by the proteasome through the action of SKP1-Cullin-F-box (SCF) E3 ligases, namely SCF^TIR1/AFB^, leading to the dissociation of Aux/IAA proteins from the ARF activators and the activation or repression of auxin response-related genes^67^,^68^,^69^. In the present study, only unigene8184_All, a ARF transcript, was down-regulated in group CK/EWAB. This suggests that unigene8184_All negatively regulates genes related to auxin. Moreover, the ubiquitin-mediated proteolysis pathway plays a central role in most hormone signaling pathways^70^, and we detected certain unigenes associated with the SCF-complex, including two up- and five down-regulated unigenes in group CK/ EWAB, three up- and five down-regulated unigenes in group CK/NPA, and three up-regulated unigenes in group EWAB/NPA (Table 4). However, we did not identify a transcriptional inhibitor similar to AUX/IAA and auxin specific targeting factor TIR1. Similarly, an AUX–IAA-ARF-mediated auxin signaling cascade is not present in microalgae, suggesting that these organisms have alternative pathways mediating auxin responses^71^. Therefore, although the mechanism itself may be conserved, it may rely on different transcriptional inhibitors recognized by an IAA-protein complex, such as AUX/IAA, that can target them for degradation. This could explain the inability of auxin to induce callus or promote AB formation, which differs from its effect on higher plants, during the process of macroalgae tissue culture.

CTK, which was detected previously in *Gracilaria sp*^72^, mediates cell division and shoot apical meristem development^73^,^74^. Therefore, CTK might play an important role in the regeneration process of *G. lichenoides* explants, as indicated by the down-regulation of nine transcripts associated with the zeatin biosynthesis pathway in group CK/EWAB, and the up-regulation of seven and six zeatin transcripts, IPT, and cytokinin dehydrogenase [EC:1.5.99.12] in groups CK/NPA and EWAB/NPA, respectively. Recently, Cheng *et al.* showed that AtIPT5 expression is negatively regulated by ARF3 through direct binding to its promoter, which affects stem cell initiation and meristem formation in *Arabidopsis thaliana*^75^. This suggests that the down-regulation of ARF genes may have led to the up-regulation of IPT genes.

Abscisic acid (ABA) is an important plant hormone that plays a role in several regulatory processes in response to abiotic stresses, and in seed dormancy, germination, and the regeneration of somatic embryos^76^,^77^,^78^,^79^,^80^,^81^. In the absence of ABA, PP2Cs (protein phosphatase 2C) directly inactivates SnRK2 (SNF1-related protein kinase) by dephosphorylating multiple residues in the kinase activation loop^82^,^83^. In the presence of ABA, PYR/PYL/RCAR (pyrabactin resistant/pyrabactin resistant-like/regulatory component of ABA Receptor), an ABA receptor, binds to and inhibits PP2Cs, resulting in the accumulation of phosphorylated SnRK2s and the subsequent phosphorylation of ABFs (ABA response element binding factors), activating downstream genes in the ABA signal transduction pathway^84^. Further, Singh *et al.* proposed that ABA, which is considered an auxin inhibitor, may reduce auxin to an optimum level required for regeneration and suppress excessive callus formation in cultures^85^. In the present study, in group CK/ EWAB, the annotated transcripts involved in the ABA signal transduction pathway included PP2C (four down-), SnRK2s (one down-), ABF (two down-regulated) transcripts, whereas no PYR/PYL/RCARs were observed. However, alternative ABA receptors may exist in *G. lichenoides*. Our results suggest that the ABA content of *G. lichenoides* explants increases during ABs formation, which reduces auxin to an optimum level to allow the formation of ABs. By contrast, in groups CK/NPA and EWAB/NPA, PP2C was up-regulated, accounting for the decrease in the ABA content of *G. lichenoides* explants when the auxin content increased after PAT was inhibited by NPA, which suppressed the induction of ABs.

Ethylene (ET) is related to the differentiation and growth of plant tissues cultured *in vitro*^86^. In the present study, CTR1, an important gene for ET signal transduction, was annotated successfully. CTR1 plays a negative regulatory role in the ET response pathway of *Arabidopsis thaliana*^87^. The ETR family of receptors directly activates the kinase activity of CTR1 in the absence of ET, which could activate CTR1 to phosphorylate downstream targets and turn off the ET response pathway. Binding of ET to the receptors inhibits their activity, resulting in the inactivation of CTR1^88^. Moreover, the formation of embryos in *Coffea canephora*is inhibited in response to inhibitors of ET synthesis (Co^2+^ ions) and ET activity (Ag^+^ ions). By contrast, exogenous ET gas partially alleviates the effect of Co^2+^ ions^86^. Furthermore, ET stimulates auxin biosynthesis and basipetal auxin transport toward the elongation zone, where it activates a local auxin response leading to inhibition of cell elongation^89^. Muday *et al.* suggested that ET modulates auxin synthesis, transport, and signaling through unique targets and responses in a range of tissues to fine tune seedling growth and development^90^. We found two annotated CTR1 unigenes that were down-regulated in group CK/EWAB, leading to the activation of ET signal transduction and enhancing the formation of ABs. However, six CTR1 unigenes were up-regulated in group CK/NPA or EWAB/NPA, indicating that ET signal transduction was suppressed because PAT was inhibited. Consequently, our results suggested that auxin and ethylene largely function synergistically to control ABs formation.

Brassinosteroids (BRs) are a group of naturally occurring novel steroidal plant hormones that regulate plant growth and development by inducing an array of physiological changes^91^,^92^,^93^. They are involved in a wide range of activities including seed germination, vascular formation, cell growth, reproductive growth, and production of flowers and fruit. BR treatment results in the elongation of excised segments of hypocotyls (seedling stems), principally because of directional cell expansion^94^. However, BRs can also promote leaf senescence^95^ and negatively regulate phototropism^96^. In the present study, seven BR biosynthesis enzymes such as det2 and DWF1were down-regulated in group CK/EWAB, indicating that BRs plays a negative role in the regeneration process of *G. lichenoidesin vitro*. One BRs biosynthesis enzyme, SMT1, was up-regulated in response to NPA, which could be attributed to auxin-mediated biosynthesis of BR. Recently, Nemhauser *et al.* proposed the transcriptional regulation of ARFs by BRs^94^. The effects of auxin and BRs on a number of Aux/IAA genes could favor the formation of specific transcriptional complexes, promoting growth. Our results indicated that auxin could suppress BRs biosynthesis, whereas BRs might bind to inhibitors of auxin and then prevent PAT, which leaded to fail to induce ABs.

The role of salicylic acid (SA) is evident in seed germination, glycolysis, flowering, fruit yield, ion uptake and transport, photosynthetic rate, stomatal conductance, and transpiration^97^. Systemic-acquired resistance (SAR) is a broad-spectrum resistance in plants that involves the up-regulation of a battery of pathogenesis-related (PR) genes^98^,^99^ and TGA, basic leucine zipper (bZIP) transcription factors, are required for the establishment of the SA-dependent SAR^100^. Several reports have described the effect of exogenous SA on enhancing somatic embryogenesis in plants^101^,^102^. In the present study, both TGA and PR1 transcripts were up-regulated to enhance the activity of defense programs in explants in response to wounding, osmotic stress, and inhibition of PAT.

The initiation of regeneration from callus may be related to the balance between different endogenous plant hormones^103^,^104^. Thus, we presumed that the mechanism of direct adventitious induction rather than callus formation of the *G. lichenoides* explants *in vitro* might be mediated by the cross-talk among different plant hormones, especially auxin.

### Antioxidant systems

Stress plays an important role during somatic embryogenesis similar to plant hormones^105^. Jin *et al.* revealed that somatic embryogenesis involves the activation of stress responses, which may regulate the balance between cell proliferation and differentiation^106^. Excised explants, which are used for callus or ABs induction in macroalgae, are subjected to sterilization procedures, cut into pieces, and cultured in an artificial nutritional environment. They therefore undergo considerable stress during this process, as indicated by the formation of reactive oxygen species (ROS). Recently, the morphological development of callus-like cells in *S.japonica* was closely linked to the activity of NADPH oxidase in the plasma membrane following ROS production^107^. Furthermore, ROS-induced signaling is entwined with plant hormonal responses. ET biosynthesis is an early O_3_ response, and later, SA and ABA are produced^108^. In contrast to the increase in signaling via the stress hormones SA, ABA, and ET, ROS treatment causes auxin signaling to be transiently suppressed. The auxin transport inhibitor TIBA causes a similar expression profile to O_3_ treatment regarding auxin-responsive genes, supporting a role for auxin transport in the regulation of apoplastic ROS responses^109^. In the present study, response to ROS was observed successfully in the GO enrichment analysis (Table 5).Genes related to ROS were mainly up-regulated by NPA, whereas SA, ABA, and BR-related genes were up-regulated. Our results suggest that ROS in combination with plant hormones, especially auxin, may provide a convenient reference to clarify the mechanism of direct formation of ABs instead of callus in the regeneration process of explants of macroalgae.

However, an inability of the explants to effectively scavenge ROS could lead to severe oxidative stress, causing damage to intracellular organelles and impairing the physiological processes related to cell regeneration. Thus, the antioxidant system, which mainly includes glutathione S-transferase (GST), NADPH, superoxide dismutase (SOD), and ascorbate peroxidase, is important for scavenging ROS during ABs formation.

GSTs are a family of genes involved in plant defense. Recently, Salvo *et al.* identified 15 maize GST genes, several of which showed an 8-fold or greater increase during early somatic embryogenesis in maize (0 to 24 h)^110^. Similarly, we observed 10, 17, and 11 GST genes with marked expression changes in groups CK/EWAB, CK/NPA, and EWAB/NPA, respectively (Table 3). More interestingly, 10 GST genes were obviously up-regulated in the EWAB/NPA group. This is consistent with the function of certain GSTs in tissue dedifferentiation, which is mediated by alterations in the redox status of the cell induced by changes in the endogenous levels of important plant growth hormones such as auxin^111^.

We found that five, seven, and four SOD unigenes showed differential expression in groups CK/EWAB, CK/NPA, and EWAB/NPA, respectively. Increased SOD activity enhances embryogenic cell differentiation during somatic embryogenesis^112^. Lin and Lai also demonstrated that a gradual increase in SOD activity during early somatic embryogenesis and late zygotic embryo development may play a key role in cell differentiation and the removal of ROS in *longan* somatic embryogenesis^113^. Therefore, variations in SOD activity might play an important role in blocking ROS production during the regeneration of *G. lichenoides* explants *in vitro*. ABs formation might be determined partially by the balance between the production and scavenging of ROS.

ROS is considered a factor controlling cell elongation in higher plants^114^. For example, rhizoid elongation in *F. serratus* may be caused by ROS produced by NADPH oxidase^115^. Moreover, NADPH oxidase shows Ca^2+^ sensitivity^116^, suggesting that high Ca^2+^ levels activate NADPH oxidase in the plasma membrane of callus-like cells and promote ROS production, resulting in cell wall loosening and cell elongation. Accumulation of ROS also occurs during multiple stages of root development, initially in response to transient cytosolic calcium increases as a result of a mechanical stimulus applied to a growing root tip^117^, and later in response to auxin during cell elongation and lateral root initiation^118^. In the present study, four, four, and two NADPH-related genes showed marked expression changes in the comparison groups CK/EWAB, CK/NPA, and EWAB/NPA, respectively. Meanwhile, one up- and fourdown-regulated genes were detected in the CK/EWAB group, while two transcripts, Unigene12429_All (11.5) and Unigene12914_All (11.3), were obviously up-regulated in response to NPA. In addition, seven calm transcripts (four up- and three down-regulated) were observed in the CK/EWAB group, whereas 14 transcripts (11 up- and three down-regulated) and 14 transcripts (nine up- and five down-regulated) were differentially expressed in CK/NPA and EWAB/NPA, respectively. It is possible that ROS production regulated by changes in calcium concentration and the activity of NADPH oxidase plays an important role in the regeneration process of explants of *G. lichenoides.*

Therefore, high levels of antioxidant activities relating to scavenging the ROS production might also determine effectively AB formation of explantsin *G. lichenoides*.

### Phosphatidylinositol (PI) signaling system

The PI metabolic pathway plays significant roles in many developmental processes, such as gene expression, hormone function, cell differentiation, cell responses to environmental factors, and interaction with other signaling pathways such as the calcium-related signaling network^119^,^120^,^121^,^122^. Connett *et al.* investigated the breakdown of membrane-bound phosphatidylinositol (PI) in homogenates of soybean (Glycine max) callus, which could be stimulated by Ca^2+^ 123. Similarly, calcium is essential for the regeneration of many plants^124^. In the present study, 13, 29, and 22 PI genes showed marked expression changes in the comparison groups CK/EWAB, CK/NPA, and EWAB/NPA, respectively, suggesting that PI signal transduction plays an important role during the formation of ABs. The addition of NPA up-regulatedcalm, PI5K, PIPK, PI(3)P5K, PLC and other PI transcripts. Further, phosphoinositide signaling has been reported to be triggered by auxin^125^. Hardtke *et al.* showed that auxin and BR signaling and biosynthesis and auxin transport might be linked by an emerging upstream connection involving calcium-calmodulin and phosphoinositide signaling^96^. Their findings together with our results suggest that PI signal transduction mediated by auxin plays an important role during formation of ABs.

**Figure 7:**
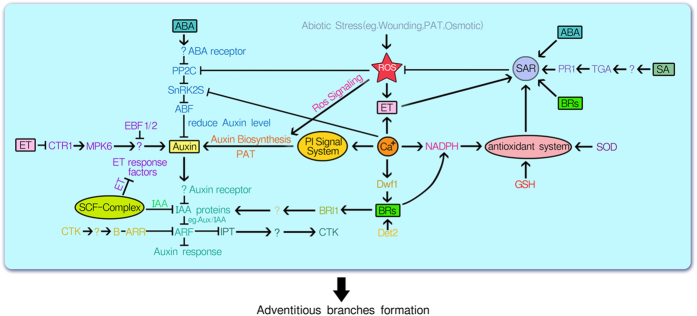
IAA-protein similar as AUX/IAA was able to suppress ARFs expression. Thus, ARFs would become active after IAA-protein was identified and degraded by SCF-complex in the presence of auxin^67^,^68^,^69^,^71^, and then suppress downstream auxin genes related to the induction of ABs. In the presence of ABA, ABA response factor such as PYR/PYL/RCARs bind to and inhibit PP2Cs, which allows the accumulation of phosphorylated SnRK2s and the subsequent phosphorylation of ABFs, leading to the activation of downstream genes of the ABA signal transduction pathway^85^. ABA is considered an auxin inhibitor, and may reduce auxin to the optimum level required for regeneration of *G. lichenoides* explants *in vitro*; CTR1 encodes a negative regulator of the ET response pathway^89^, which might modulate auxin synthesis, transport, and signaling with unique targets and responses resulting in AB formation. Auxin could suppress BR biosynthesis, whereas BR binds to inhibitor proteins of auxin, resulting inthe prevention of PAT. ARFs could directly bind to the promoter of IPT and negatively regulate IPT expression^75^, affecting AB formation. The type-B ARRs act as transcription factors and their phosphorylation activates the transcription of the cytokinin-regulated genes, which negatively regulate auxin signaling^126^. ROS-induced signaling is entwined with plant hormonal responses. Systemic-acquired resistance (SAR) is a broad-spectrum resistance in plants that involves the up-regulation of a battery of pathogenesis-related (PR) genes^100^,^101^ and basic leucine zipper (bZIP) transcription factors TGA are required for the establishment of the SA-dependent plant defense response systemic-acquired resistance^102^. ET biosynthesis is an early O_3_ response, and later, SA and ABA are produced^110^. In contrast to the increase in signaling via the stress hormones SA, ABA, and ET, ROS treatment caused auxin signaling to be transiently suppressed. The auxin transport inhibitor TIBA gave a similar expression profile toO_3_ treatment for auxin-responsive genes, supporting a role for auxin transport in the regulation of apoplastic ROS responses^111^. The antioxidant system mainly includes GST, NADPH, and SOD, which are very important for scavenging the ROS production, which effectively promotes AB formation; auxin and brassinosteroid signaling and biosynthesis and auxin transport might be linked by an emerging upstream connection involving calcium–calmodulin and phosphoinositide signaling^96^ during explant regeneration of *G. lichenoides in vitro*.

In conclusion, the mechanism of direct AB induction rather than callus formation in *G. lichenoides* explants *in vitro* is complex and may be mediated by cross-talk among multiple elements, including plant hormones, antioxidant systems and other signal transduction pathways (Fig. 7). We investigated the mechanism of macroalgae tissue culture using the Illumina sequencing platform for the first time and identified important genomic factors that could help clarify the process of regeneration of macroalgae in the future.

## Additional Information

**How to cite this article**: Wang, W. *et al.* Transcriptome analysis identifies genes involved in adventitious branch formation of  *Gracilaria lichenoides in vitro.* *Sci. Rep.* **5**, 17099; doi: 10.1038/srep17099 (2015).

## Figures and Tables

**Table 1:** Output statistics of sequencing.

Samples	Total Raw Reads	Total Clean Reads	Total Clean Nucleotides(nt)	Q20 percentage	N percentage	GC percentage
CK	80,114,686	73,618,690	6,625,682,100	97.53%	0.00%	52.30%
EWAB	74,467,532	68,469,170	6,162,225,300	97.54%	0.00%	51.99%
NPA	61,857,640	57,050,510	5,134,545,900	97.67%	0.00%	51.77%

^*^Total reads and total nucleotides are actually clean reads and clean nucleotides. The total nucleotides should be greater than contract provision. The Q20percentage is the proportion of nucleotides with a quality value higher than 20. The N percentage is the proportion of unknown nucleotides in clean reads. The GC percentage is the proportion of guanidine and cytosine nucleotides among total nucleotides.

**Table 2:** Statistics of assembly quality.

	Sample	Total Number	Total length(nt)	Mean length(nt)	N50
Contig	CK	31,366	22,560,219	719	2851
	EWAB	16,454	19,027,454	1156	3558
	NPA	20,119	19,455,379	967	3328
Unigene	CK	21,362	31,928,105	1495	4188
	EWAB	13,424	30,635,306	2282	4704
	NPA	14,955	29,811,145	1993	4353
	All	21,294	40,091,203	1883	5055

^*^N50: length of the smallest transcripts in the set that contain the fewest (largest).

**Table 3:** Transcripts related to auxin according to the GO enrichment analysis.

GO ID	GO NAMES	Transcripts numbers (up-, down regulated numbers)		
		CK/EWAB	CK/NPA	EWAB/NPA
GO:0009734	auxin mediated signaling pathway	7 (1 up, 6 down)	8 (2 up, 6 down)	4 (2 up, 2 down)
GO:0009733	response to auxin stimulus	24 (1 up, 23 down)	28 (7 up, 21 down)	8 (6 up, 2 down)
GO:0060918	auxin transport	10 (10 down)	11 (1 up, 10 down)	1 (1 up)
GO:0009850	auxin metabolic processes	5 (5 down)	5 (1 up, 4 down)	1 (1 up)
GO:0009851	auxin biosynthetic processes	3 (3 down)	4 (1 up, 3 down)	1 (1 up)

^*^In this table and the following tables, “B/A” always means B is control and A is treated.

**Table 4:** KEGG pathways with differentiallyexpressed immune-related gene enrichment in different groups

Pathway	Number of unigenes (up, down-regulation)		
	CK/EWAB	CK/NPA	EWAB/NPA
ABCB1 transporter	20 (1 up, 19 down)	22 (5 up, 17 down)	6 (5 up, 1 down)
**Plant hormone signal transduction**	**28 (28 down)**	**35 (9 up, 26 down)**	**17 (17 up)**
*AUXIN*	*1 (1 down)*	*1 (1 down)*	*0*
ARF	1 (1 down)	1 (1 down)	0
*ZEATIN*	*9 (9 down)*	*11 (7 up, 8 down)*	*6 (6 up)*
IPT	9 (9 down)	11 (3 up, 8 down)	2 (2 up)
cytokinin dehydrogenase	0	4 (4 up)	4 (4 up)
*ABA*	*7 (7 down)*	*9 (2 up, 7 down)*	*2 (2 up)*
PP2C	4 (4 down)	5 (1 up, 4 down)	1 (1 up)
SnRK2	1 (1 down)	2 (1 up, 1 down)	1 (1 up)
ABF	2 (2 down)	2 (2 down)	0
*ETHYLENE*	*5 (5 down)*	*9 (6 up, 3 down)*	*6 (6 up)*
CTR1	2 (2 down)	6 (6 up)	6 (6 up)
MPK6	3 (3 down)	3 (3 down)	0
*SALICYLIC ACID*	*0*	*1 (1 up)*	*2 (2 up)*
TGA	0	0	1 (1 up)
PR-1	0	1 (1 up)	1 (1 up)
*Brassinosteroid biosynthesis*	*7 (7 down)*	*8 (1 up, 7 down)*	*1 (1 up)*
DET 2	1 (1 down)	1 (1 down)	0
STE 1	2 (2 down)	2 (2 down)	0
SMO 1	1 (1 down)	1 (1 down)	0
CYP51G1	1 (1 down)	1 (1 down)	0
SMO 2	1 (1 down)	1 (1 down)	0
DWF 1	1 (1 down)	1 (1 down)	0
SMT 1	0	1 (1 up)	1 (1 up)
**Ubiquitin mediated proteolysis**	**52 (6 up, 46 down)**	**55 (11 up, 44down)**	**12 (11 up, 1 down)**
*E1*	*4 (4down)*	*5 (1 up, 4 down)*	*1 (1 up )*
*E2*	*18 (3 up, 15 down)*	*20 (4 up, 16 down)*	*5 (4 up, 1 down)*
*E3*	*30 (3 up, 27down)*	*30 (6 up, 24 down)*	*6 (6 up)*
SCF complex	7 (2 up, 5 down)	8 (3 up, 5 down)	3 (3 up)
others	23(1 up, 22 down)	22 (3 up, 19down)	3 (3 up)
**Antioxidant systems**	**33 (7 up, 26 down)**	**45(17 up, 28 down)**	**21 (18 up, 3 down)**
Superoxide dismutase	5 (1 up, 4 down)	7 (3 up, 4 down)	4 (3 up, 1 down)
Glutathione S-transferase	10 (4 up, 6 down)	17 (11 up, 6 down)	11 (10 up, 1 down)
NADPH	4 (1 up, 3 down)	4 (2 up, 2 down)	2 (2 up)
Ascorbate peroxdase	6 (6 down)	5 (1 up, 4 down)	1 (1 up)
Catalase	6 (6 down)	6 (6 down)	0
PRDX5	2 (1 up, 1 down)	6 (6 down)	3 (2 up, 1 down)
**Phosphatidylinositol signaling system**	**13 (4 up, 9 down)**	**29 (19 up,10 down)**	**22 (17 up, 5 down)**
calm	7 (4 up, 3 down)	14 (11 up,3 down)	14 (9 up, 5 down)
PI5K	0	2 (2 up)	2 (2 up)
PIPK	0	3 (3 up)	3 (3 up)
PLC	1 (1 down)	3 (1 up, 2 down)	1 (1 up)
PI(3)P5K	0	2 (2 up)	2 (2 up)
OTHERS	5 down	5 down	0

**Table 5:** Transcripts related to ROS by GO enrichment analysis.

GO ID	GO NAMES	Transcripts numbers (up-, down regulated numbers)		
		CK/EWAB	CK/NPA	EWAB/NPA
GO:0000302	response to reactive oxygen species	32 (10 up, 22 down)	41 (20 up, 21 down)	23 (18 up, 5 down)
GO:0072593	reactive oxygen species metabolic process	19 (3 up, 16 down)	23 (7 up, 16 down)	6 (5 up, 1 down)
GO:2000377	regulation of reactive oxygen species metabolic process	5 (5 down)	6 (2 up, 4 down)	2 (2 up)
ALL		48 (10 up, 38 down)	56 (22 up, 34 down)	26 (20 up, 6 down)

## References

[b1] Gao S. *et al.* A Strategy for the Proliferation of *Ulva prolifera,* MainCausative Species of Green Tides, with Formation of Sporangia by Fragmentation. PloS One, 5, e8571 (2010). 20052408 10.1371/journal.pone.0008571 PMC2797376

[b2] Gao S., Shen S. D., Wang G. C., Niu J. F., Lin A. P. & Pan G. H. PSI-Driven Cyclic Electron Flow Allows Intertidal Macro-Algae *Ulva* sp. (*Chlorophyta*) to Survive in Desiccated Conditions. Plant Cell Physiol. 52, 885–893 (2011). 21471121 10.1093/pcp/pcr038

[b3] Yang R. L., Zhou W., Shen S. D., Wang G. C., He L. W. & Pan G. H. Morphological and photosynthetic variations in the process of spermatia formation from vegetative cells in *Porphyra yezoensis* Ueda (*Bangiales, Rhodophyta*) and their responses to desiccation. Planta, 235, 885–893 (2012). 22101945 10.1007/s00425-011-1549-y

[b4] Gao S. *et al.* The physiological links of the increased photosystem II activityin moderately desiccated *Porphyra haitanensis* (*Bangiales, Rhodophyta*) to the cyclic electron flow during desiccation and re-hydration. Photosynth. Res. 116, 45–54 (2013). 23896795 10.1007/s11120-013-9892-4

[b5] Wang W. L. *et al.* Exploring valid internal-control genes in *Porphyra yezoensis* (*Bangiaceae*) during stress response conditions. Chinese Journal of Oceanology and Limnology 32, 783–791 (2014).

[b6] Schramm W. Cultivation of unattached seaweeds. Seaweed resources in Europe, uses and potential (ed. Guiry M. D. & Blunden G. ) 379–408 (Wiley, New York, 1991).

[b7] Armisen R. World-wide use and importance of *Gracilaria*. J. Appl. Phycol. 7, 231–243 (1995).

[b8] Bixler H. J. & Porse H. A decade of change in the seaweed hydrocolloids industry. J. Appl. Phycol. 23, 321–335 (2011).

[b9] Xu Y. J., Fang J. G. & Wei W. Application of *Gracilaria lichenoides (Rhodophyta)* for alleviating excess nutrients in aquaculture. J. Appl. Phycol. 20, 199–203 (2008).

[b10] Roberts D. A., Paul N. A., Dworjanyn S. A., Bird M. I. & Nys R. D. Biochar from commercially cultivated seaweed for soil amelioration. Sci. Rep. 5, 9665 (2015). 25856799 10.1038/srep09665 PMC4391317

[b11] Stevens D. R. & Purton S. Genetic engineering of eucaryotic algae: progress and prospects. J. Phycol. 33, 713–722 (1997).

[b12] Yokoya N. S. & Yoneshigue-Valentin Y. Micropropagation as a tool for sustainable utilization and conservation of populations of *Rhodophyta*. Braz. J. Pharmacogn. 21, 334–339 (2011).

[b13] Gusev M. V., Tambiev A. H., Kirikova N. N., Shelyastina N. N. & Aslanyan R. R. Callus formation in seven species of agarophyte marine algae. Mar. Biol. 95, 593–597 (1987).

[b14] Kaczyna F. & Megne,t W. H. The effects of glycerol and plant growth regulators on *Gracilaria verrucosa (Gigartinales, Rhodophyceae)*. Hydrobiologia 268, 57–64. (1993).

[b15] Huang W. & Fujita Y. Callus induction and thallus regeneration in some species of red algae. Phycol. Res. 45, 105–111 (1997).

[b16] Yokoya N. S., Kakita H., Obika H. & Kitamura T. Effects of environmental factors and plant growth regulators on growth of the red alga *Gracilaria vermiculophylla* from Shikoku Island, Japan. Hydrobiologia 398/399, 339–347 (1999).

[b17] Yokoya N. S. Apical callus formation and plant regeneration controlled by plant growth regulators on axenic culture of red alga *Gracilariopsis tenuifrons (Gracilariales, Rhodophyta)*. Phycol. Res. 48, 133–142 (2000).

[b18] Collantes G., Melo C. & Candia A. Micropropagation by explants of *Gracilaria chilensis Bird, McLachlan and Oliveira Gloria*. J. Appl. Phycol. 16, 203–213 (2004).

[b19] Yokoya N. S., West J. A. & Luchi A. E. Effects of plant growth regulators on callus formation, growth and regeneration in axenic tissue cultures of *Gracilaria tenuistipitata* and *Gracilaria perplexa (Gracilariales, Rhodophyta)*. Phycol. Res. 52, 244–254 (2004).

[b20] Kumar G. R., Reddy C. R. K. & Jha B. Callus induction and thallus regeneration from callus of phycocolloid yielding seaweeds from the Indian coast. J. Appl. Phycol. 19, 15–25 (2007).

[b21] Ramlov F., Plastino E. M. & Yokoya N. S. Growth, callus formation and plant regeneration in color morphs of *Gracilaria domingensis (Gracilariales, Rhodophyta)* cultured under different irradiance and plant growth regulators. Phycologia 52(6), 508–516 (2013).

[b22] Chen C. S., Zhang J. R. & Lin A. F. Tissue culture for five species of *Gracilariales in vitro* (in Chinese). J. Fujian Fisheries 2, 19–24 (1987).

[b23] Tan E. L. Tissue culture and protoplast isolation of *Gracilaria changii (Rhodophyta)*. Master’s thesis, University of Malaya, Kuala Lumpur (1999).

[b24] Xie X. J., Wang G. C., Pan G. H., Gao S., Xu P. & Zhu J. Y. Variations in morphology and PSII photosynthetic capabilities during the early development of tetraspores of *Gracilaria vermiculophylla* (Ohmi) Papenfuss (*Gracilariales, Rhodophyta*). BMC Developmental Biol. 10, 43 (2010). 10.1186/1471-213X-10-43 PMC2873408 20426804

[b25] Yeong H. Y., Phang S. M., Reddy C. R. K. & Khalid N. Production of clonal planting materials from *Gracilaria changii* and *Kappaphycus alvarezii* through tissue culture and culture of *G. changii* explants in airlift photobioreactors. J. Appl. Phycol. 26, 729–746 (2014).

[b26] Reddy C. R. K., Jha B., Fujita Y. & Ohno M. Seaweed micropropagation techniques and their potentials: an overview. J. Appl. Phycol. 20, 619–632 (2008b).

[b27] Baweja P., Sahoo D., García-Jiménez P. & Robaina R. R. Seaweed tissue culture as applied to biotechnology: Problems, achievements and prospects. Phycol. Res. 57, 45–58 (2009).

[b28] Reddy C. R. K., Gupta V. & Jha B. Developments in biotechnology of red algae. Red algae in genomic age (ed. Chapman D. J. & Seckbach J. ) 307–341 (Springer Sci. Business Media, New York, 2010).

[b29] Trewavas A. J. Growth substance sensitivity: the limiting factor in plant development. Plant Physiol. 55, 60–72 (1982).

[b30] Lipperheide M. A., Francisco J., Rodríyuez E. & Evans L. V. Facts, problems, and needs in seaweed tissue culture: an appraisal. J. Phycol. 31, 677–688 (1995).

[b31] Yokoya N. S. *et al.* Endogenous cytokinins, auxins and abscisic acid in red algae from Brazil. J. Phycol. 46, 1198–1205 (2010).

[b32] Ying H. *et al.* Auxin-induced WUS expression is essential for embryonic stem cell renewal during somatic embryogenesis in Arabidopsis. Plant J. 59, 448–460 (2009). 19453451 10.1111/j.1365-313X.2009.03880.x PMC2788036

[b33] Su Y. H. *et al.* Auxin-induced WUS expression is essential for embryonic stem cell renewal during somatic embryogenesis in Arabidopsis. Plant J. 59, 448–460 (2009). 19453451 10.1111/j.1365-313X.2009.03880.x PMC2788036

[b34] Henrichs S. *et al.* Regulation of ABCB1/PGP1-catalysed auxin transport by linker phosphorylation. EMBOI J. 31, 2965–2980 (2012). 10.1038/emboj.2012.120 PMC3395086 22549467

[b35] Ozsolak F. & Milos P. M. RNA sequencing: advances, challenges and opportunities. Nat. Rev. Genet. 12, 87–98 (2011) 21191423 10.1038/nrg2934 PMC3031867

[b36] Van Verk M. C., Hickman R., Pieterse C. M. J. & Van Wees S. C. M. RNA-Seq: revelation of the messengers. Trends Plant Sci. 18, 175–179 (2013). 23481128 10.1016/j.tplants.2013.02.001

[b37] Xu J. Y. *et al.* Comparative analysis of four essential *Gracilariaceae* species in China based on whole transcriptomic sequencing. Acta Oceanol. Sin. 33, 54–62 (2014).

[b38] Guillard R. R. L. Culture of phytoplankton for feeding marine invertebrates. *Culture of marine invertebrate animals* (ed. Smith W. L. & Chanley M. H. ) 29–60 (Plenum, New York, 1975).

[b39] Shen S. D., Wu X. J., Yan B. L. & He L. H. Tissue culture of three species of *Laurencia* complex. Chin. J. Oceanol. Limnol. 28, 514–520 (2010).

[b40] Chan C. X., Teo S. S., Ho C. L., Othman R. Y. & Phang S. M. Optimisation of RNA extraction from *Gracilaria changii (Gracilariales, Rhodophyta)*. J. Appl. Phycol. 16, 297–301 (2004).

[b41] Brent Ewing. & Phil Green. Base-Calling of Automated Sequencer TracesUsingPhred. II. Error Probabilities. Genome Res. 8, 186–194 (1998). 9521922

[b42] Grabherr M. G. *et al.* Full-length transcriptome assembly from RNA-Seq data without a reference genome. Nat. Biotechnol. 29, 644–652 (2011). 21572440 10.1038/nbt.1883 PMC3571712

[b43] Ali M., Williams A., McCue K., Schaeffer L. & Wold B. Mapping and quantifying mammalian transcriptomes by RNA-Seq. Nat. Methods 5, 621–628 (2008). 18516045 10.1038/nmeth.1226 PMC13303166

[b44] Iseli C., Jongeneel C. V. & Bucher P. ESTScan: a program for detecting, evaluating, and reconstructing potential coding regions in EST sequences. Proceeding of the Seventh International Conference on Intelligent Systems for Molecular Biology (ed. Lengauer T., Schneider R., Bork P., Brutlag D., Glasgow J., Mewes H. W. & Zimmer R. ) 138–148 (AAAI, CA, 1999). 10786296

[b45] Conesa A. *et al.* Blast2GO: a universal tool for annotation, visualization and analysis in functional genomics research. Bioinformatics 21, 3674–3676 (2005). 16081474 10.1093/bioinformatics/bti610

[b46] Ye J. *et al.* WEGO: a web tool for plotting GO annotations. Nucleic Acids Res. 34, W293–W297 (2006). 16845012 10.1093/nar/gkl031 PMC1538768

[b47] Kanehisa M. *et al.* KEGG for linking genomes to life and the environment. Nucleic Acids Res. 36, D480–D484 (2006). 18077471 10.1093/nar/gkm882 PMC2238879

[b48] Moriya Y., Itoh M., Okuda S., Yoshizawa A. C. & Kanehisa M. KAAS: an automatic genome annotation and pathway reconstruction server. Nucleic Acids Res. 35, W182–W185 (2007). 17526522 10.1093/nar/gkm321 PMC1933193

[b49] Audic S. & Claverie J. M. The significance of digital gene expression profiles. Genome. Res. 7, 986–995 (1997). 9331369 10.1101/gr.7.10.986

[b50] Thiel T., Michalek W. & Varshney R. K. Graner Exploiting EST databases for the development and characterization of gene-derived SSR-markers in barley (Hordeum vulgareL.) Theor. Appl. Genet. 106, 411–422 (2003). 12589540 10.1007/s00122-002-1031-0

[b51] Hagen G. The control of gene expression by auxin. *Plant Hormones-Physiology, Biochemistry and Molecular Biology* (ed. Davies P. J. ) 228–245 (Kluwer Academic Publishers, Dordrecht, 1995).

[b52] Grossman A. R. Paths towards algal genomics. Plant Physiol. 137, 410–427 (2005). 15710682 10.1104/pp.104.053447 PMC1065345

[b53] Walker T. L., Collet C. & Purton S. Algal transgenic in the genomic era. J. Phycol. 41, 1077–1093 (2005).

[b54] Flintoft L. Transcriptomics: Measuring gene expression in non-model organisms. Nat. Rev. Genet. 12, 742 (2011).

[b55] Muers M. Gene expression: Transcriptome to proteome and back to genome. Nat. Rev. Genet. 12, 518 (2011). 21709688 10.1038/nrg3037

[b56] Mravec J. *et al.* Interaction of PIN and PGP transport mechanisms in auxin distribution-dependent development. Development 135, 3345–3354 (2008). 18787070 10.1242/dev.021071

[b57] Bail A. L. *et al.* Auxin Metabolism and Function in the Multicellular Brown Alga *Ectocarpus siliculosus*. Plant Physiol. 153, 128–144 (2010). 20200071 10.1104/pp.109.149708 PMC2862433

[b58] Murphy A. S., Hoogner K. R., Peer W. A. & Taiz L. Identification, purification, and molecular cloning of N-1-naphthylphthalmic acid-binding plasma membrane-associated aminopeptidases from *Arabidopsis*. Plant Physiol. 128, 935–950 (2002). 11891249 10.1104/pp.010519 PMC152206

[b59] Geisler M. *et al.* Cellular efflux of auxin catalyzed by the *Arabidopsis* MDR/PGP transporter AtPGP1. Plant J. 44, 179–194 (2005). 16212599 10.1111/j.1365-313X.2005.02519.x

[b60] Rojas-Pierce M. *et al.* Arabidopsis P-Glycoprotein19 Participates in the Inhibition of Gravitropism by Gravacin. Chem. Biol. 14, 1366–1376 (2007). 18096505 10.1016/j.chembiol.2007.10.014

[b61] Nagashima A., Uehara Y. & Sakai T. The ABC Subfamily B Auxin Transporter AtABCB19 is Involved in the Inhibitory Effects of N-1-Naphthyphthalamic Acid on the Phototropic and Gravitropic Responses of *Arabidopsis* Hypocotyls. Plant Cell Physiol. 49(8), 1250–1255 (2008). 18556728 10.1093/pcp/pcn092

[b62] Kim J. Y. *et al.* Identification of an ABCB/P-glycoprotein-specific inhibitor of auxin transport by chemical genomics. J. Biol. Chem. 285, 23309–23317 (2010). 20472555 10.1074/jbc.M110.105981 PMC2906323

[b63] Bailly A. *et al.* Modulation of P-glycoproteins by auxin transport inhibitors is mediated by interaction with immunophilins. J. Biol. Chem. 283, 21817–21826 (2008). 18499676 10.1074/jbc.M709655200

[b64] Lomax T. L., Muday G. K. & Rubery P. H. Auxin transport. Plant Hormones: Physiology, Biochemistry and Molecular Biology (ed. Davies P. J. ) 509–530 (Kluwer Academic Publishers, Dordrecht, 1995).

[b65] Luschnig C. Auxin transport: why plants like to think BIG. Curr. Biol. 11, R831–R833 (2001). 11676938 10.1016/s0960-9822(01)00497-3

[b66] Guilfoyle T. J. & Hagen G. Auxin response factors. Curr. Opin. Plant Biol. 10, 453–460 (2007). 17900969 10.1016/j.pbi.2007.08.014

[b67] Gray W. M., Kepinski S., Rouse D., Leyser O. & Estelle M. Auxin regulates SCF TIR1-dependent degradation of Aux/IAA proteins. Nature 414, 271–276 (2001). 11713520 10.1038/35104500

[b68] Tiwari S. B., Hagen G. & Guilfoyle T. The roles of auxin response factor domains in auxin-responsive transcription. Plant Cell 15, 533–543 (2003). 12566590 10.1105/tpc.008417 PMC141219

[b69] Villalobos C. *et al.* A combinatorial TIR1/AFB-Aux/IAA co-receptor system for differential sensing of auxin. Nat. Chem. Biol. 18, 477–485 (2012). 10.1038/nchembio.926 PMC3331960 22466420

[b70] Santner A. & Estelle M. Recent advances and emerging trends in plant hormone signaling. Nature 459, 1071–1078 (2009). 19553990 10.1038/nature08122

[b71] Lau S., Shao N., Bock R., Jürgens G. & Smet I. D. Auxin signaling in algal lineages: fact or myth? Trends Plant Sci. 14, 182–188 (2009). 19285905 10.1016/j.tplants.2009.01.004

[b72] Stirk W. A. *et al.* Cytokinins in macroalgae. Plant Growth Regul. 41, 13–24 (2003).

[b73] Kerstetter R. A. & Hake S. Shoot meristem formation in vegetative development. Plant Cell 9, 1001–1010 (1997). 12237372 10.1105/tpc.9.7.1001 PMC156974

[b74] Leibfried A. *et al.* WUSCHEL controls meristem function by direct Regulation of cytokinin-inducible response regulators. Nature 438, 1172–1175 (2005). 16372013 10.1038/nature04270

[b75] Cheng Z. J. *et al.* Pattern of Auxin and Cytokinin Responses for Shoot Meristem Induction Results from the Regulation of Cytokinin Biosynthesis by AUXIN RESPONSE FACTOR 3. Plant Physiol. 161, 240–251 (2013). 23124326 10.1104/pp.112.203166 PMC3532255

[b76] Hilhorst H. W. M. & Karssen C. M. Seed dormancy and germination: the role of abscisic acid and gibberellins and the importance of hormone mutants. Plant Growth Regul. 11, 225–238 (1992).

[b77] Rohde A., Kurup S. & Holdsworth M. ABI3 emerges from the seed. Trends Plant Sci. 5, 418–419 (2000b). 11203275 10.1016/s1360-1385(00)01736-2

[b78] Xiong L. M. & Zhu J. K. Regulation of Abscisic Acid Biosynthesis. Plant Physiol. 133, 29–36 (2003). 12970472 10.1104/pp.103.025395 PMC523868

[b79] Mondal T. K., Bhattacharya A., Sood A. & Ahuja P. S. Factors affecting germination and conversion frequency of somatic embryos of Tea [*Camellia sinensis* (*L*.) *O. Kuntze*]. J. Plant Physiol. 159, 1317–1321 (2002).

[b80] Sharma P., Pandey S., Bhattacharya A., Nagar P. K. & Ahuja P. S. ABA associated biochemical changes during somatic embryo development in *Camellia sinensis* (*L.*) *O. Kuntze*. J. Plant Physiol. 161, 1269–1276 (2004). 15602818 10.1016/j.jplph.2004.01.015

[b81] Hauser F., Waadt R. & Schroeder J. I. Evolution of Abscisic Acid Synthesis and Signaling Mechanisms. Curr. Biol. 21, R346–R355 (2011). 21549957 10.1016/j.cub.2011.03.015 PMC3119208

[b82] Umezawa T. *et al.* Type 2C protein phosphatases directly regulate abscisic acid-activated protein kinases in Arabidopsis. Proc. Natl. Acad. Sci. USA 106, 17588–17593 (2009). 19805022 10.1073/pnas.0907095106 PMC2754379

[b83] Vlad F. *et al.* Protein Phosphatases 2C Regulate the Activation of the Snf1-Related Kinase OST1 by Abscisic Acid in Arabidopsis. Plant Cell 21, 3170–3184 (2009). 19855047 10.1105/tpc.109.069179 PMC2782292

[b84] Cutler S. R., Rodriguez P. L., Finkelstein R. R. & Abrams S. R. Abscisic acid: emergence of a core signaling network. Annu. Rev. Plant. Biol. 61, 651–679 (2010). 20192755 10.1146/annurev-arplant-042809-112122

[b85] Singh A., Jani k., Sagervanshi A. & Agrawal P. K. High-Frequency Regeneration by Abscisic Acid (ABA) from Petiole Callus of *Jatropha curcas*. In Vitro Cell Dev. Biol. Plant 50, 638–645 (2014).

[b86] Hatanaka T., Sawabe E., Azuma T., Uchida N. & Yasuda T. The role of ethylene in somatic embryogenesis from leaf discs of *Coffea canephora*. Plant Sci. 107, 199–204 (1995).

[b87] Huang Y., Li H., Hutchison C. E., Laskey J. & Kieber J. J. Biochemical and functional analysis of CTR1, a protein kinase that negatively regulates ethylene signaling in *Arabidopsis*. Plant J. 33, 221–233 (2003). 12535337 10.1046/j.1365-313x.2003.01620.x

[b88] Mantiri F. R. *et al.* The transcription factor MtSERF1 of the ERF subfamily identified by transcriptional profiling is required for somatic embryogenesis induced by auxin plus cytokinin in *Medicago truncatula*. Plant Physiol. 146, 1622–1636 (2008). 18235037 10.1104/pp.107.110379 PMC2287338

[b89] Ruzicka K. *et al.* Ethylene regulates root growth through effects on auxin biosynthesis and transport-dependent auxin distribution. Plant cell 19, 2197–2212 (2007). 17630274 10.1105/tpc.107.052126 PMC1955700

[b90] Muday G. K., Rahman A. & Binder B. M. Auxin and ethylene: collaborators or competitors? Trends Plant Sci. 17, 181–195 (2012). 22406007 10.1016/j.tplants.2012.02.001

[b91] Topping J. F., May V. J., Muskett P. R. & Lindsey K. Mutations in the HYDRA1 gene of Arabidopsis perturb cell shape and disrupt embryonic and seedling morphogenesis. Development 124, 4415–4424 (1997). 9334289 10.1242/dev.124.21.4415

[b92] Diener A. C. *et al.* Sterol methyltransferase 1 controls the level of cholesterol in plants. Plant Cell 12, 853–870 (2000). 10852933 10.1105/tpc.12.6.853 PMC149089

[b93] Khripach V., Zhabinskii V. & de Groot A. Twenty years of brassinosteroids: steroidal plant hormones warrant better crops for the XXI century. Ann. Bot. 86, 441–447 (2000).

[b94] Nemhauser J., Mockler T. C. & Chory J. Interdependency of brassinosteroid and auxin signaling in *Arabidopsis*. PloS Biol. 2, e258 (2004). 15328536 10.1371/journal.pbio.0020258 PMC509407

[b95] Clouse S. D. & Sasse J. M. BRASSINOSTEROIDS: essential regulators of plant growth and development. Annu. Rev. Plant Physiol. Plant Mol. Biol. 49, 427–451 (1998). 15012241 10.1146/annurev.arplant.49.1.427

[b96] Hardtke C. S., Dorcey E., Osmont K. S. & Sibout R. Phytohormone collaboration: zooming in on auxin–brassinosteroid interactions. Trends Cell Biol. 17, 485–492 (2007). 17904848 10.1016/j.tcb.2007.08.003

[b97] Khan W., Prithiviraj B. & Smith D. L. Photosynthetic responses of corn and soybean to foliar application of salicylates. J. Plant Physiol. 160, 485–492 (2003). 12806776 10.1078/0176-1617-00865

[b98] Zhang Y., Fan W., Kinkema M., Li X. & Dong X. Interaction of NPR1 with basic leucine zipper protein transcription factors that bind sequences required for salicylic acid induction of the PR1gene. Proc. Natl. Acad. Sci. USA 96, 6523–6528 (1999). 10339621 10.1073/pnas.96.11.6523 PMC26915

[b99] Kinkema M., Fan W. & Dong X. Nuclear localization of NPR1 is required for activation of PR gene expression. Plant Cell 12, 2339–2350 (2000). 11148282 10.1105/tpc.12.12.2339 PMC102222

[b100] Zander M., Camera S. L., Lamotte O., Metraux J. P. & Gatz C. *Arabidopsis thaliana* class-II TGA Transcription factors are essential activators of jasmonic acid/ethylene-induced defense responses. Plant J. 61, 200–210 (2010). 19832945 10.1111/j.1365-313X.2009.04044.x

[b101] Pius J., George L., Eapen S. & Rao P. S. Enhanced plant regeneration in pearl millet (*Pennisetum americanum*) by ethylene inhibitors and cefotaxime. Plant Cell Tiss. Org. 32, 91–96 (1993).

[b102] Luo J. P., Jiang S. T. & Pan L. J. Enhanced somatic embryogenesis by salicylic acid of *Astragalus adsurgens* Pall: relationship with H_2_O_2_ production and H_2_O_2_-metabolizing enzyme activities. Plant Sci. 161, 125–132 (2001).

[b103] Sugiyama M. Organogenesis *in vitro.* Curr. Opin. Plant Biol. 2, 61–64 (1999). 10047565 10.1016/s1369-5266(99)80012-0

[b104] Zhang Y. F., Zhou J. H., Wu T. & Cao J. S. Shoot regeneration and the relationship between organogenic capacity and endogenous hormonal contents in pumpkin. Plant Cell Tiss. Org. 93, 323–331 (2008).

[b105] Karami O. & Saidi A. The molecular basis for stress-induced acquisition of somatic embryogenesis. Mol. Biol. Rep. 37, 2493–2507 (2010). 19705297 10.1007/s11033-009-9764-3

[b106] Jin F. Y. *et al.* Comparative transcriptome analysis between somatic embryos (SEs) and zygotic embryos in cotton: evidence for stress response functions in SE development. Plant Biotechnol. J. 12, 161–173 (2014). 24112122 10.1111/pbi.12123

[b107] Kanamori M., Mizuta H. & Yasui H. Effects of ambient calcium concentration on morphological form of callus-like cells in *Saccharina japonica* (*Phaeophyceae*) sporophyte. J. Appl. Phycol. 24, 701–706 (2012). 10.1007/s10811-011-9750-8 PMC3434887 23002326

[b108] Overmyer K., Brosché M. & Kangasjärvi J. Reactive oxygen species and hormonal control of cell death. Trends Plant Sci. 8, 335–342 (2003). 12878018 10.1016/S1360-1385(03)00135-3

[b109] Blomster T. *et al.* Apoplastic Reactive Oxygen Species Transiently Decrease Auxin Signaling and Cause Stress-Induced Morphogenic Response in *Arabidopsis*. Plant Physiol. 157, 1866–1883 (2011). 22007024 10.1104/pp.111.181883 PMC3327221

[b110] Salvo S. A. G. D., Hirsch C. N., Buell C. R., Kaeppler S. M. & Kaeppler H. F. Whole Transcriptome Profiling of Maize during Early Somatic Embryogenesis Reveals Altered Expression of Stress Factors and Embryogenesis-Related Genes. PloS One 9, e111407 (2014). 25356773 10.1371/journal.pone.0111407 PMC4214754

[b111] Feher A., Pasternak T. P. & Dudits D. Transition of somatic plant cells to an embryogenic state. Plant Cell Tiss. Org. 74, 201–228 (2003).

[b112] Cui K. R., Xing G. S., Liu X. M., Xing G. M. & Wang Y. F. Effect of hydrogen peroxide on somatic embryogenesis of *Lycium barbarum* L. Plant Sci. 146, 9–16 (1999).

[b113] Lin Y. L. & Lai Z. X. Superoxide dismutase multigene family in longan somatic embryos: a comparison of CuZn-SOD, Fe-SOD, and Mn-SOD gene structure, splicing, phylogeny, and expression. Mol. Breeding 32, 595–615 (2013).

[b114] Passardi F., Penel C. & Dunand C. Perfoming the paradoxical: how plant peroxidases modify the cell wall. Trends Plant Sci. 9, 534–540 (2004). 15501178 10.1016/j.tplants.2004.09.002

[b115] Coelho S. M. B., Brownlee C. & Bothwell J. H. F. A tip-high, Ca^2+^ -interdependent, reactive oxygen species gradient is associated with polarized growth in *Fucus serratus* zygotes. Planta 227, 1037–1046 (2008). 18087716 10.1007/s00425-007-0678-9

[b116] Kobayashi M. *et al.* Calcium-dependent protein kinase regulate the promotion of reactive oxygen species by potato NADPH oxidase. Plant Physiol. 19, 1065–1080 (2007). 10.1105/tpc.106.048884 PMC1867354 17400895

[b117] Monshausen G. B., Bibikova T. N., Weisensee,l M. H. & Gilroy S. Ca^2+^ regulates reactive oxygen species production and pH during mechanosensing in *Arabidopsis* roots. Plant Cell 21, 2341–2356 (2009). 19654264 10.1105/tpc.109.068395 PMC2751959

[b118] Joo J. H. *et al.* Auxin-induced reactive oxygen species production requires the activation of phosphatidylinositol 3-kinase. FEBS Lett. 579, 1243–1248 (2005). 15710420 10.1016/j.febslet.2005.01.018

[b119] Munnik T., Irvine R. F. & Musgrave A. Phospholipid signaling in plants. Biochim. Biophys. Acta 1389, 222–72 (1998). 9512651 10.1016/s0005-2760(97)00158-6

[b120] Berridge M. J., Bootman M. D. & Lipp P. Calcium-a life and death signal. Nature 395, 645–648 (1998). 9790183 10.1038/27094

[b121] Carafoli E. Calcium signaling: A tale for all seasons. Proc. Natl. Acad. Sci. USA 99, 1115–1122 (2002). 11830654 10.1073/pnas.032427999 PMC122154

[b122] Lin W. H., Ye R., Ma H., Xu Z. H. & Xue H. W. DNA chip-based expression profile analysis indicates involvement of the phosphatidylinositol signaling pathway in multiple plant responses to hormone and abiotic treatments. Cell Res. 14, 34–45 (2004). 15040888 10.1038/sj.cr.7290200

[b123] Connett R. J. A. & Hanke D. E. Breakdown of phosphatidylinositol in soybean callus. Planta 169, 216–221 (1986). 24232553 10.1007/BF00392317

[b124] Jain P. & Rashid A. Stimulation of shoot regeneration on *Linum* hypocotyls segments by thidiazuron and its response to light and calcium. Biol. Plantarum 44, 611–613 (2001).

[b125] Ettlinger C. & Lehle L. Auxin induces rapid changes in phosphatidylinositol metabolites. Nature 331, 176–178 (1988). 3257546 10.1038/331176a0

[b126] Moubayidin L., Mambro R. D. & Sabatini S. Cytokinin–auxin crosstalk. Trends Plant Sci. 14, 557–562 (2009). 19734082 10.1016/j.tplants.2009.06.010

