# Corrigendum: Transcriptome analysis identifies genes involved in adventitious branches formation of *Gracilaria lichenoides in vitro*

**DOI:** 10.1038/srep20457

**Published:** 2016-02-03

**Authors:** Wenlei Wang, Huanqin Li, Xiangzhi Lin, Shanjun Yang, Zhaokai Wang, Baishan Fang

Scientific Reports 5: Article number: 1709910.1038/srep17099; published online: 12112015; updated: 02032016

In the original version of this Article, there were errors in Affiliation 1 which was incorrectly listed as ‘College of Biochemistry and Engineering, Xiamen University, Xiamen 361005, China.’ The correct affiliation is listed below:

Department of Chemical and Biochemical Engineering, College of Chemistry and Chemical Engineering, Xiamen University, Xiamen, Fujian, 361005, China

These errors have now been corrected in the PDF and HTML versions of the Article.

